# Identification and evaluation of *BAG* (B-cell lymphoma-2 associated athanogene) family gene expression in pigeonpea (*Cajanus cajan*) under terminal heat stress

**DOI:** 10.3389/fgene.2024.1418380

**Published:** 2024-11-14

**Authors:** Chakravaram Alekhya, Avuthu Tejaswi, Gadeela Harika, Naresh Bomma, Prakash I. Gangashetty, Wricha Tyagi, Kalenahalli Yogendra

**Affiliations:** Research Program-Accelerated Crop Improvement, International Crops Research Institute for the Semi-Arid Tropics, Hyderabad, India

**Keywords:** BAG genes, climate resilience, heat stress, pigeonpea, signaling

## Abstract

**Introduction:**

Heat stress poses a significant environmental challenge, impacting plant growth, diminishing crop production, and reducing overall productivity. Plants employ various mechanisms to confront heat stress, and their ability to survive hinges on their capacity to perceive and activate appropriate physiological and biochemical responses. One such mechanism involves regulating multiple genes and coordinating their expression through different signaling pathways. The *BAG* (B-cell lymphoma-2 associated athanogene) gene family plays a multifunctional role by interacting with heat shock proteins, serving as co-chaperones, or regulating chaperones during the response to heat stress and development. While numerous studies have explored BAG proteins in model plants, there still remains a knowledge gap concerning crop plants.

**Methods:**

Our study successfully identified nine *BAG* genes in pigeonpea through genome-wide scanning. A comprehensive in silico analysis was conducted to ascertain their chromosomal location, sub-cellular localization, and the types of regulatory elements present in the putative promoter region. Additionally, an expression analysis was performed on contrasting genotypes exhibiting varying heat stress responses.

**Results:**

The results revealed eight *CcBAG* genes with higher expression levels in the tolerant genotype, whereas *BAG6 (Cc_02358)* exhibited lower expression. Upstream sequence analysis identified *BAG* members potentially involved in multiple stresses.

**Discussion:**

The functional characterization of these *BAG* genes is essential to unravel their roles in signaling pathways, facilitating the identification of candidate genes for precise breeding interventions to produce heat-resilient pigeonpea.

## Introduction

Plant productivity on a global scale faces a significant threat from heat stress, leading to notable reductions in crop yields. The adverse impacts of heat stress on the growth and development of plants are varied and can manifest at different stages of their life cycle (Zhao et al., 2020). In particular, legumes seem sensitive to heat stress during the blooming stage. Exposure to elevated temperatures ranging from 30°C to 35°C for a few days can severely impact key reproductive processes, including microsporogenesis, megasporogenesis, fertilization, and early embryogenesis. This can result in damaged pods and significant losses in yield (Mohapatra et al., 2020; Liu et al., 2019). Furthermore, heat stress negatively impacts various physiological aspects, including photosynthesis, respiration, membrane stability, the production of reactive oxygen species (ROS), and the accumulation of antioxidants (Djanaguiraman et al., 2020; Hasanuzzaman et al., 2013). It is important to note that heat stress is often compounded by other abiotic stresses like salt or drought, but understanding the independent mechanisms by which heat stress affects plant growth and metabolism is crucial for effectively mitigating the combined effects of these stresses.

Plants have developed a range of mechanisms to confront heat stress, allowing them not only to survive but also thrive in high-temperature surroundings. These mechanisms can be broadly classified into avoidance and tolerance strategies (Bita and Gerats, 2013; Cao et al., 2023). The specific coping mechanisms of a plant depend on its species and the prevailing environmental conditions. In response to heat stress, plants exhibit distinctive cellular and molecular reactions involving alterations in cell structure organization and membrane functions aimed at enduring challenging conditions. This period triggers an up-regulation of genes responsible for molecular chaperones and signaling molecules that orchestrate events in response to heat stress (Boopathy et al., 2022; Ul Haq et al., 2019).

One particular set of co-chaperones involved in these responses are BAG (B-cell lymphoma-2 associated athanogene) proteins, which regulate processes like protein folding, cellular signaling, translocation, and degradation during heat stress (Arif et al., 2021; Nawkar et al., 2017). BAG proteins are evolutionarily conserved, showcasing multifunctional properties that contribute to cell survival in scenarios such as apoptosis and stress responses (Kabbage and Dickman, 2008; Wang et al., 2020; Huang et al., 2022). A distinctive feature of BAG proteins is the conserved BAG domain (BD) located at their C-terminus, which interacts with the ATPase domain of heat shock protein 70 (HSP70). HSP70 is a molecular chaperone critical for folding and unfolding proteins, playing a pivotal role in shielding cells from damage (Doukhanina et al., 2006).

Within the plant kingdom, the BAG protein family can be categorized into two groups based on their structural characteristics, determined through genome-wide alignment and conservative domain identification (Jiang et al., 2023; Kabbage and Dickman, 2008). The first group possesses an N-terminal ubiquitin-like (UBL) domain, while the second group features a plant-specific CaM-binding IQ motif near the BAG domain (Doukhanina et al., 2006; Dash and Ghag, 2022). Despite the known roles of BAG proteins in responding to abiotic and biotic stresses in plants, limited research has explored their specific involvement in the response to heat stress.

The *AtBAG*4 gene in *Arabidopsis* exhibited increased expression in response to heat stress, and it subsequently interacted with HSP70 to prevent cell death induced by the stress. A heat-shock element within the AtBAG5 promoter was also identified to respond to heat induction (Nawkar et al., 2017). In maize, heat stress led to the upregulating of 13 genes belonging to the *BAG* family (Li-Zong et al., 2013). The potential BAG gene, *HSG1*, in grapes, demonstrated heat shock-inducible properties. The overexpression of *HSG1* in *Arabidopsis* resulted in enhanced heat tolerance, even at extremely high temperatures (Kobayashi et al., 2012). Additionally, *TaBAG2*, identified as a stress-inducible gene in wheat, conferred thermotolerance when overexpressed in *Arabidopsis* (Ge et al., 2016). On the contrary, the *BAG* gene from tomato, specifically the *SlBAG9* gene, which is a homolog of *AtBAG*5, exhibited negative regulation concerning heat tolerance. Overexpressing *SlBAG9* led to the development of tomato plants that are highly sensitive and susceptible to heat stress (Ding et al., 2022).

Pigeonpea (*Cajanus cajan*) is a leguminous crop known for its high protein content. It is a significant provider of essential nutrients such as proteins, vitamins, and minerals in human diets and cattle feed (Kuraz Abebe, 2022). However, the productivity of this crop has stagnated in recent decades due to the detrimental impact of abiotic stresses in the evolving climate. The cumulative consequences of heat stress on pigeonpea result in diminished yield and altered crop quality. The degree of yield reduction varies depending on factors such as the growth stage, intensity, and duration of heat stress. Although the involvement of *BAG* genes in responding to heat stress has been documented in various plants like *Arabidopsis* (Doukhanina et al., 2006), tomato (Ding et al., 2022), wheat (Ge et al., 2016), and maize (Li-Zong et al., 2013), there is currently a lack of comprehensive genome-wide phylogenetic and functional studies on *BAG* genes specifically for pigeonpea. However, the recent availability of high-quality genomic data and the increasing research focus on climate change, particularly heat stress, have highlighted significant environmental challenges that impact pigeonpea growth and production. These developments have prompted us to prioritize functional genomic studies, including identifying gene families such as *BAG*, which are known to be involved in heat stress responses in Arabidopsis.

To gain a deeper insight into the evolutionary dynamics of *BAG* genes in pigeonpea and uncover their potential contributions to heat stress response, we identified nine *CcBAG* genes in pigeonpea. Through *in silico* analyses, we documented various aspects, including their chromosomal locations, motif compositions, *cis*-acting elements, evolutionary relationships, genomic collinearity, and selection pressure. Additionally, we conducted an expression analysis of these *BAG* genes under heat stress conditions. These findings offer valuable insights into the functions of *BAG* family members and present opportunities for utilizing them in the development of heat-tolerant pigeonpea varieties.

## Materials and methods

### Identification and sequence analysis of the pigeonpea *BAG* gene family

To identify *BAG* genes in pigeonpea, an exhaustive search was conducted using the coding sequences of seven *BAG* genes from *Arabidopsis thaliana*, sourced from The *Arabidopsis* Information Resource (TAIR) at (https://www.arabidopsis.org/). These genes served as blast queries applied to the pigeonpea genomic sequence data available on the Legume Information System (LIS) database (https://www.legumeinfo.org/). Using the physical location information retrieved from the LIS database, all identified pigeonpea *BAG* genes were subsequently mapped to chromosomes with the assistance of the MapGene2Chromosome (MG2C) tool (Version 2.1, accessible at (http://mg2c.iask.in/mg2c_v2.1/). The molecular weight, theoretical isoelectric point (pI), aliphatic index and grand average of hydropathicity (GRAVY) values of the *BAG* genes were extracted using the Expasy protparam tool (https://web.expasy.org/protparam/) (Gasteiger et al., 2005). The *in silico* analysis workflow for the pigeonpea *BAG* gene family is summarised in Supplementary Figure S1.

### Phylogenetic analysis and sub-cellular localization

A total of 49 amino acid sequences representing BAG family proteins from *C. cajan, Glycine max, Medicago truncatula, Arachis hypogea, and Cicer arietinum* were extracted from the LIS database using the coding sequences of the *BAG* genes from *A. thaliana* as a template. The amino acid sequences of *Arabidopsis* were retrieved from the TAIR database. These amino acid sequences underwent alignment through the MUSCLE algorithm, and a phylogenetic tree was constructed using MEGA 11.0, employing the neighbour-joining method and a bootstrap test involving 1,000 replications (Tamura et al., 2021). Additionally, the subcellular localization of pigeonpea BAG family proteins was predicted *in silico* using the BUSCA tool, accessible at (https://busca.biocomp.unibo.it/) (Savojardo et al., 2018).

### Domain analysis, gene structure analysis

BAG-conserved domains were generated using the HMMER web server (Bio sequence analysis using profile hidden Markov Models) (Potter et al., 2018) (https://www.ebi.ac.uk/Tools/hmmer/). The presence of these domains in the BAG proteins was further validated using the Interpro scan tool (https://www.ebi.ac.uk/interpro/search/sequence/). To visualize the exon-intron structures of the *BAG* genes, coding and genome sequences were employed in conjunction with the Gene Structure Display Server software (http://gsds.gao-lab.org/) (Hu et al., 2015).

### Collinearity and evolutionary analysis of identified *BAG* genes

*Medicago truncatula*, recognized as a model legume plant, displays a substantial abundance of widely conserved regions with pigeonpea, surpassing other legumes. Due to these distinctive characteristics, *M. truncatula* was selected as the model for conducting genome collinearity and evolutionary analyses of *BAG* genes in pigeonpea. The analysis was carried out using the Multiple Collinearity Scan toolkit (MCScanX) program integrated into TBtools, with specific parameters set as follows: CPU for BLASTp (2), Number of BLAST hits (5), and E-value (1e^−10^). The multiple synteny plotter tool within TBtools was employed to identify orthologous genes in the organisms, visually connecting orthologous *BAG* genes of pigeonpea with *M. truncatula* using curved arches. To determine the non-synonymous substitution rates and synonymous substitution rates of the identified orthologous gene pairs, the Ka/Ks calculator tool in TBtools version 2.0 was utilized (Chen et al., 2023).

### *Cis*-regulatory element analysis of the identified *BAG* genes

To identify the *cis*-regulatory elements within the promoter region of the identified *BAG* genes, a 2,000-bp upstream region from the transcriptional start site for each gene was acquired from Cajanus Mine (https://mines.legumeinfo.org/cajanusmine/begin.do). The identification of *cis*-regulatory elements involved, submitting the obtained upstream sequences to the PlantCARE online tool (Lescot et al., 2002) (http://bioinformatics.psb.ugent.be/webtools/plantcare/html/). The visualization of the results from the *cis*-regulatory element analysis was conducted using TBtools.

### Protein-protein interaction analysis and 3D structure prediction of *CcBAG* protein

To predict the potential protein-protein interaction (PPI) network, the identified pigeonpea BAG proteins were submitted to the STRING online tool (Szklarczyk et al., 2015) (http://string-db.org). By utilizing BLAST, the orthologs of these proteins were recognized in tomato, and the ortholog with the most substantial bit score was subsequently examined in greater detail. Any predicted proteins that did not exhibit interactions were excluded from further consideration. Understanding the 3D structure of proteins helps us to gain insights into their functions, mechanisms, and interactions. Hence, the 3D structure of *CcBAG*s was predicted by the PHYER2 server (Lovell et al., 2002) (www.phyre2/html). Moreover, the I-TASSER server (Zhang, 2008) was used to verify the models. These structures were further visualized through the UCSF Chimera visualization tool (Pettersen et al., 2004).

### GO and miRNA prediction of *CcBAG* genes

To explore the potential functions of candidate target genes, BLAST2GO (https://www.blast2go.com/) analysis was performed. The GO annotations were categorized into three distinct groups: cellular component, molecular function, and biological process. Also, the miRNA sequences for the *BAG* genes were obtained from the Plant microRNA Encyclopedia (PmiREN) database (https://pmiren.com/database). To identify the target sites of the retrieved miRNA sequences in these genes, psRNATarget (Dai et al., 2018) (https://www.zhaolab.org/psRNATarget/) was utilized. The visualization of the predicted target sites was then done using the Geneious Prime software package v2024.0.

### Expression analysis of *BAG* genes during terminal heat stress

#### Plant material

The selfed progenies of two medium-duration pigeonpea (ICPL 87119 and ICPL 85063 - used as heat susceptible genotypes) and one mid-early genotype (TS3R-used as heat tolerant genotype) were used in this study. Pigeonpea, being inherently sensitive to both photoperiod and temperature, served as the subject of the study. To assess sensitivity to photoperiod and thermotolerance, two sets of progenies were utilized. One set consisted of flowering progenies from each genotype, while the other set comprised non-flowering progenies from the same genotypes during the summer of 2023. Notably, ICPL87119 and ICPL85063 are recognized for their heightened sensitivity to both photoperiod and temperature. In contrast, TS3R demonstrates a high level of tolerance to heat stress, particularly during the summer season.

The pigeonpea genotypes were cultivated in the deep black soils at the International Crops Research Institute for the Semi-Arid Tropics (ICRISAT) in Patancheru, Hyderabad, India (Latitude: 17.51°N, Longitude: 78.27°E, Altitude: 545 m). Planting commenced on 27 January 2023. Due to their photosensitivity, some progenies of ICPL 87119, ICPL 85063, and TS3R did not flower. On 21 May 2023, at 2 p.m., we collected flowers and leaves from the flowering plants, as well as leaf samples from the non-flowering plants. These samples were collected under a maximum recorded temperature of 40°C (Supplementary Table S1), with the plants having been exposed to temperatures ranging from 35°C to 40°C for 40–45 days. These samples were then used for expression analysis.

#### Gene expression analysis

For the quantitative reverse-transcription polymerase chain reaction (qRT-PCR), we isolated total RNA from flower and leaf samples of divergent pigeonpea genotypes exposed to heat stress using the RNeasy Plant Mini kit (Qiagen, Tokyo, Japan) with three replications. A 2.0 μg portion of purified RNA underwent cDNA synthesis following the recommended protocol (Thermoscript RT-PCR system, Invitrogen, Carlsbad, CA, United States). The quantitative PCR was performed using gene-specific primers (Supplementary Table S2) on a CFX96TM Real-Time System (Bio-Rad, India). To normalize cycle threshold (Ct) values, the pigeonpea GAPDH (XM_020358085) gene served as the housekeeping gene (Sinha et al., 2015). The qRT-PCR reaction conditions were set in triplicates as follows: 2 min at 95°C, 40 cycles of 10 s at 95°C, and 20 s at 60°C. Additionally, a fluorescence measurement at each 0.5°C variation from 60°C to 95°C over 20 min was included to obtain the melting curve. The relative fold expression was calculated using the 2^−ΔΔCt^ method (Livak and Schmittgen, 2001).

## Results

### Identification and sequence analysis of the pigeonpea *BAG* gene family

The *Arabidopsis BAG* genes were used as query sequences to search for their orthologs against the pigeonpea in the LIS database (Nawkar et al., 2017). A total of 12 genes were initially retrieved, and subsequent analysis using InterProScan and manual inspection led to the removal of redundant or false-positive genes. Ultimately, nine non-redundant *CcBAG* genes were identified in pigeonpea, comprising 2 *BAG1* (*Cc_18023* and *Cc_26867*), 1 *BAG2* (*Cc_19692*), 4 *BAG4* (*Cc_15012*, *Cc_11905*, *Cc_01922*, and *Cc_16501*), 1 *BAG5* (*Cc_19448*), and 1 *BAG6* (*Cc_02358*).

These nine *CcBAG* proteins exhibited molecular weights ranging from 19.41 to 129.75 kDa, and their corresponding amino acid lengths varied between 172 and 1,156 (Supplementary Table S3). The predicted theoretical isoelectric points (pI) for *Cc_15012*, *Cc_01922*, *Cc_16501*, *Cc_19448*, and *Cc_02358* proteins fell within the acidic range, while the pI for the remaining proteins (*Cc_18023*, *Cc_26867*, *Cc_19692*, and *Cc_11905*) were in the basic range. The aliphatic index ranged from 55.47 to 101.56 indicating the thermostability of these CcBAG proteins. It was 101.56 for the gene *Cc_19448* predicting it to be the most thermostable, while *Cc_02358* identified to be the least with a value of 55.47. Further, the GRAVY values predicted for these *BAG* genes were all negative ranging from −0.062 (*Cc_19448)* to −1.092 (*Cc_02358)*, indicating those to be hydrophilic. Additional important properties, including the length of the coding region (CDS), the number of exons/introns, chromosomal location, and cellular localization of the identified genes, were investigated to enhance our understanding of *BAG* genes in pigeonpea (Supplementary Table S3).

**FIGURE 1:**
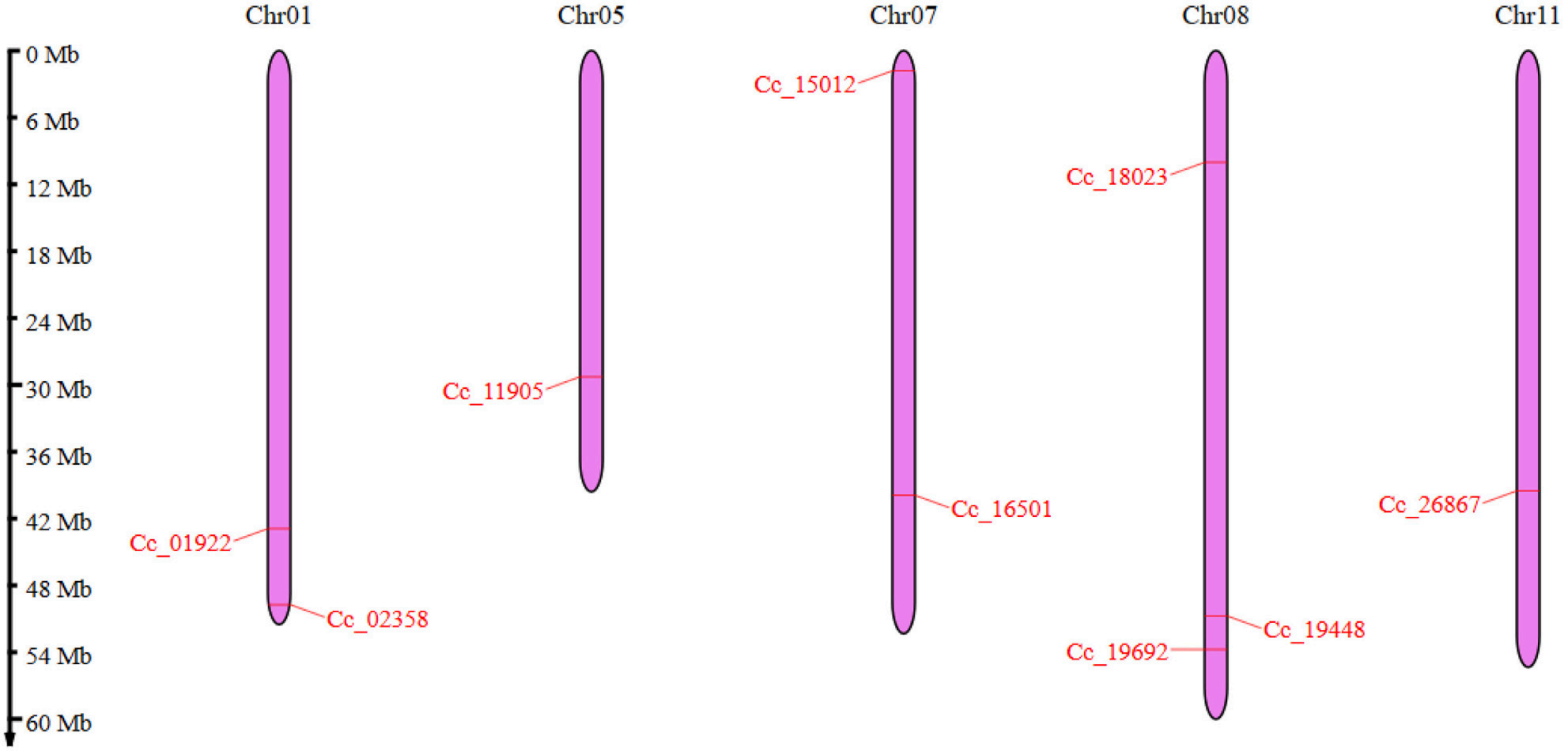
Chromosomal locations of *BAG* genes in pigeonpea. The chromosomes are represented in purple color and the genes are shown in red colored lines. The Scale on the left side represents the length of the chromosome in Mb.

The *CcBAG* genes identified were spread across five chromosomes in pigeonpea: Chr01, Chr05, Chr07, Chr08, and Chr11. Notably, Chr08 contained three distinct *CcBAG* genes (*Cc_18023, Cc_19448, Cc_19692*). Chr01 included the genes *Cc_01922* and *Cc_02358*, Chr07 had *Cc_15012* and *Cc_16501*, and Chr11 and Chr05 housed the genes *Cc_26867* and *Cc_11905*, respectively (Figure 1).

### Phylogenetic analysis and sub-cellular localization

**FIGURE 2:**
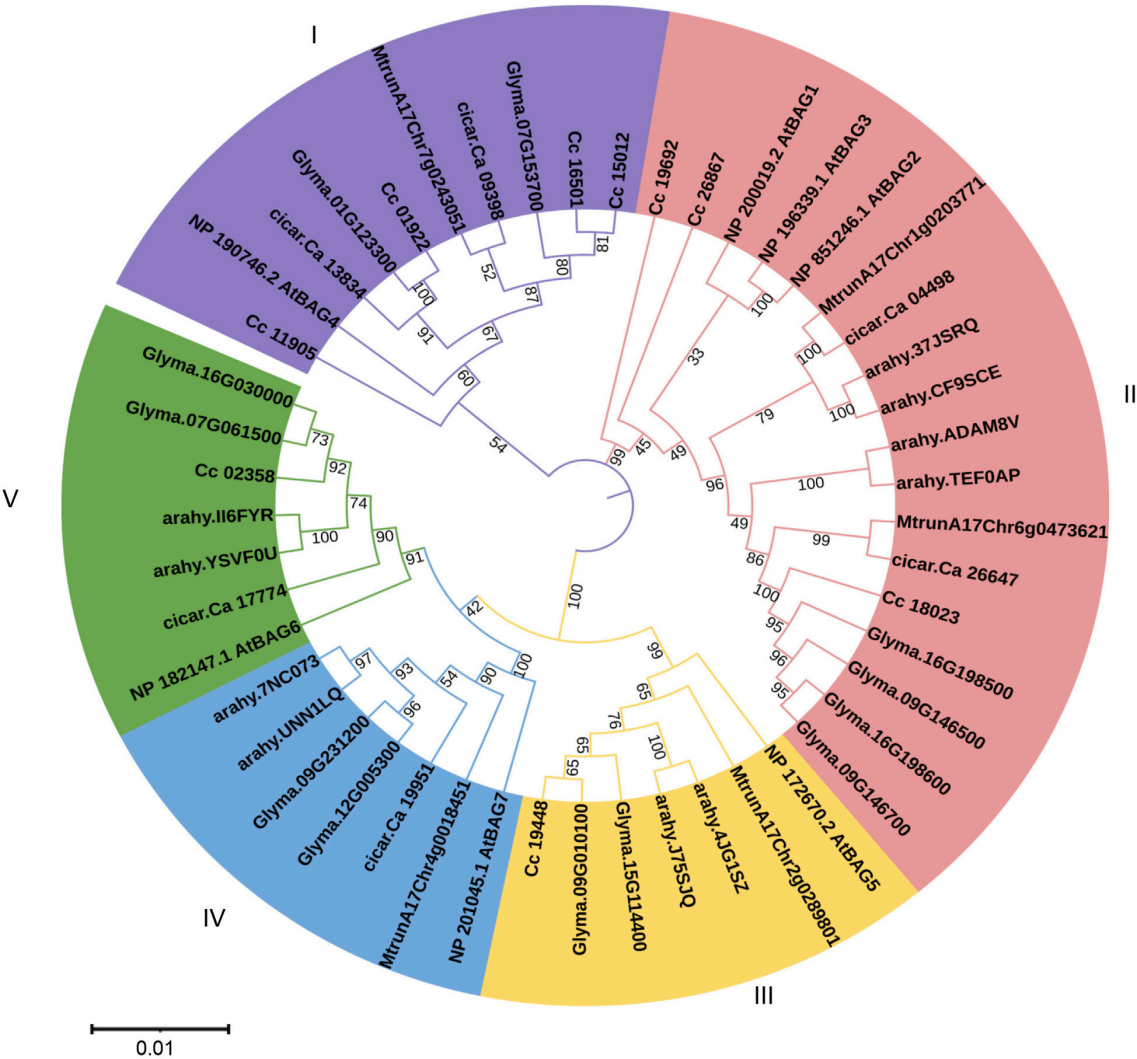
Phylogenetic analysis of *BAG* genes of *Arabidopsis*, pigeonpea, chickpea, soybean, medicago, and groundnut. Five clades are represented in five colors. Trees are constructed using the Neighbor-Joining method with 1,000 bootstrap replications in MEGA X software.

To identify the phylogenetic relationships among pigeonpea BAG proteins implicated in the regulation of heat stress, a neighbour-joining phylogenetic tree was constructed. This analysis encompassed BAG proteins from *C. cajan* (9 genes), *A. thaliana* (7 genes), *G. max* (12 genes), *M. truncatula* (5 genes), *A. hypogea* (10 genes), and *C. arietinum* (6 genes) (Figure 2). In total, 49 genes were organized into five distinct clades labelled as I to V. Among these clades, clade II exhibited the highest membership with 18 members, followed by clade I with 10 members, and clades III, IV, and V, each comprising 7 members.

The *CcBAG* proteins were examined for their anticipated subcellular localization using the BUSCA (Bologna Unified Subcellular Component Annotator) tool. The outcomes revealed that out of the nine proteins, seven *CcBAG*s were predicted to be localized in the nucleus, with the exception of *Cc_26867* (*BAG1*) and *Cc_19448* (*BAG5*), which were predicted to be situated in the chloroplast (Supplementary Table S3).

### Collinearity and evolutionary analysis of identified *BAG* genes

**FIGURE 3:**
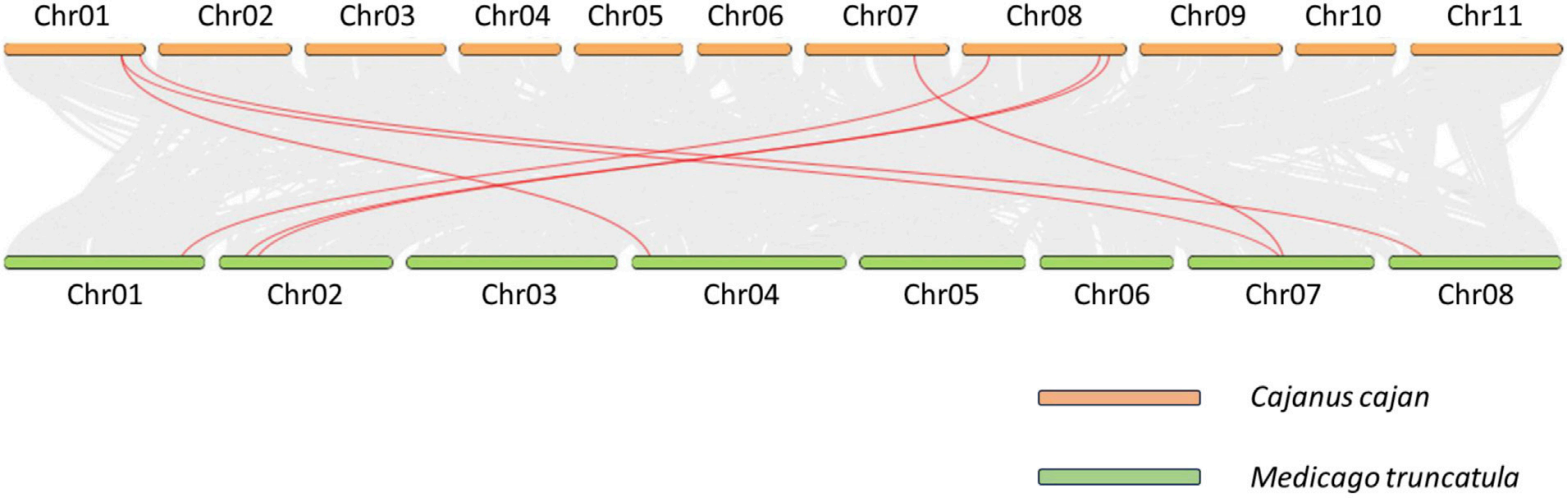
Collinearity analysis of *BAG* genes. Orthologous collinear *BAG* genes of *Cajanus* cajan and *Medicago* truncatula are represented by red curved lines on a gray background.

To better understand the phylogenetic connections among pigeonpea *BAG* genes and other members of the Fabaceae family, we conducted a collinearity analysis involving *M. truncatula* (Figure 3). Among the 9 *CcBAG* genes identified in pigeonpea, only 7 exhibited collinear genes in *M. truncatula*, with *Cc_26867* and *Cc_11905* lacking collinear counterparts in the considered species.

The identification of the synonymous to non-synonymous mutation ratio (Ka/Ks) aids in understanding the selection pressure acting on identified gene pairs in pigeonpea (Supplementary Table S4). Among the 9 genes, only 6 (*Cc_18023*, *Cc_01922*, *Cc_15012*, *Cc_16501*, *Cc_19448*, and *Cc_02358*) displayed significant collinearity with Medicago and were subsequently subjected to Ka/Ks analysis (Wang et al., 2013; Hyun et al., 2014). The calculated Ka/Ks values ranged from 0.07 to 0.45, all of which are less than 1. This suggests that these six *CcBAG* genes underwent purifying or stabilizing selection pressure throughout their evolutionary history.

### Domain analysis and gene structure analysis

**FIGURE 4:**
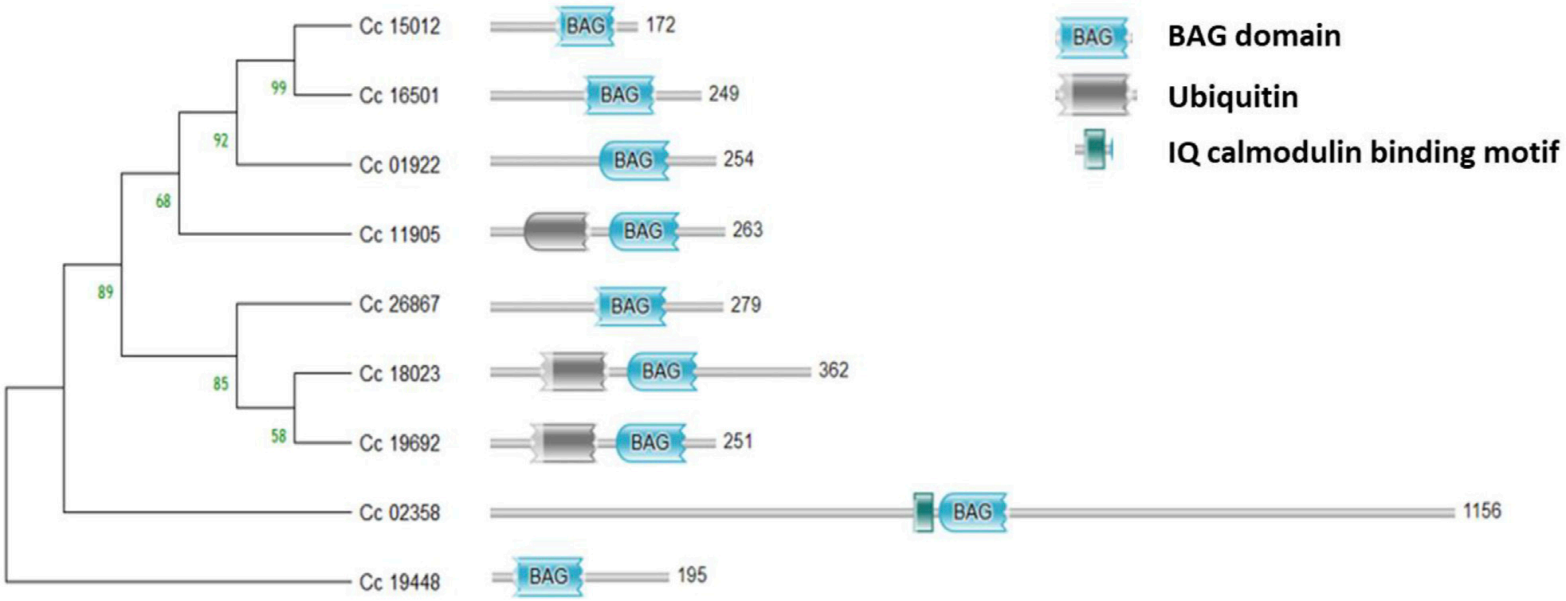
Domain analysis of the identified *BAG* genes showing the conserved *BAG* domain in the C-terminus, Ubiquitin domain in the N-terminus, and IQ calmodulin-binding domain.

**FIGURE 5:**
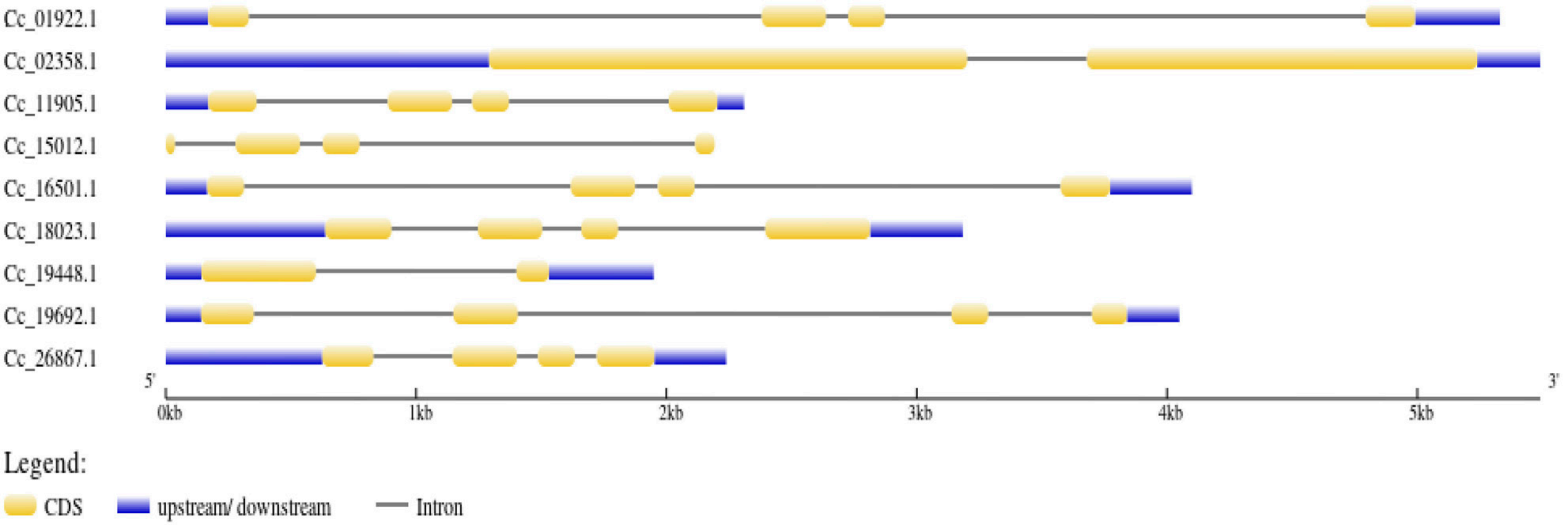
Gene structure representation of *BAG* genes. Exons are represented as yellow boxes; introns are represented as black lines while untranslated regions (UTRs) are represented as blue boxes.

The *BAG* domain (BD) located in the C-terminus of the genes was identified using the HMMER web server. Additionally, the genes *Cc_18023* (*BAG1*), *Cc_19692* (*BAG2*) in group II, and *Cc_11905* (*BAG4*) in group I were found to contain UBL domains in addition to the BAG domain. Conversely, *Cc_02358* (*BAG6*) in group V featured an IQ calmodulin-binding motif situated in the N-terminus of the gene (Figure 4). In the case of *CcBAG*s, the number of exons ranged from 2 to 4. All genes in Clades I and II possessed four exons, while those in Clades III and V had only two exons (Figure 5). These findings suggest a high level of evolutionary conservation among members of the pigeonpea *BAG* gene family.

### *Cis*-regulatory element analysis of the identified *BAG* genes

**FIGURE 6:**
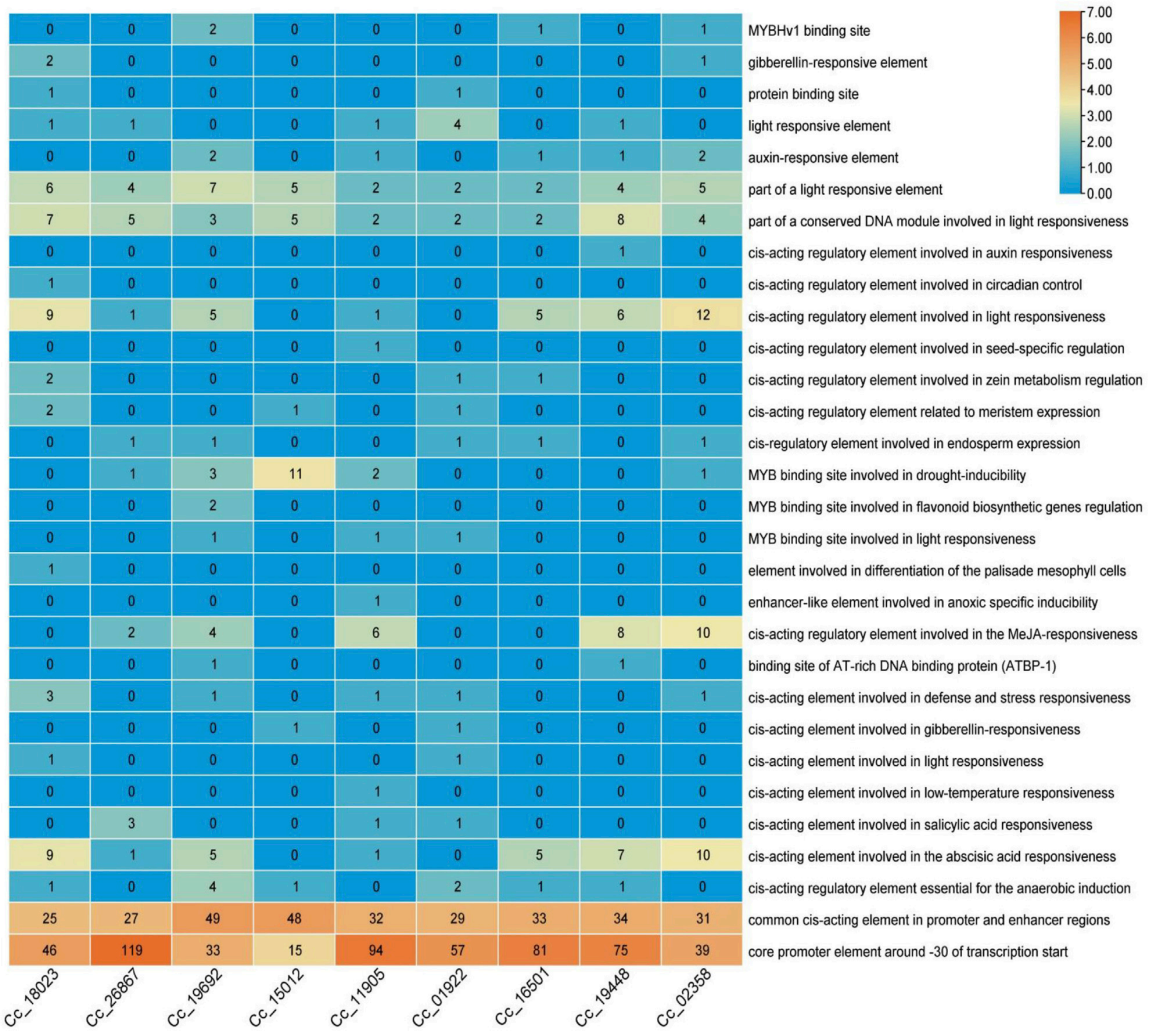
*Cis*-regulatory elements analysis of *BAG* genes. The scale on the right represents the color scale based on the number of *cis*-regulatory elements.

To further analyze the potential regulatory mechanisms of *CcBAG* genes, *cis*-elements associated with the putative promoter regions of these genes were identified by extracting the 2,000 bp upstream sequence from the transcription start site and analysed for *cis*-regulatory elements (Figure 6).

The findings revealed the presence of shared upstream elements responsive to various environmental stimuli, such as light, defense, stress, low-temperature, and drought. Specifically, cis-acting elements involved in defense and stress responsiveness, *cis*-acting elements involved in low temperature responsiveness are observed, with elements for light responsiveness noted high in *BAG5* (*Cc_19448*), and 1 *BAG6* (*Cc_02358*). Additionally, elements associated with anaerobic and anoxic induction, along with different binding sites for AT-rich DNA proteins and MYB (myeloblastosis-related proteins) involved in drought inducibility caused by heat stress that are specifically high in *BAG4* (*Cc_15012)*, were identified. Furthermore, several elements responsive to hormones like gibberellin, auxin, methyl jasmonic acid (MeJA), abscisic acid, and salicylic acid, as well as growth-related elements like circadian control, endosperm expression, and elements involved in the differentiation of palisade mesophyll cells were observed. In particular, elements involved in MeJA responsiveness and abscisic acid responsiveness, which are possible hormone-regulated pathways in heat stress mechanism, are seen exceptionally in BAG5 (Cc_19448) and 1 BAG6 (Cc_02358). The occurrence and quantity of these elements in the upstream regions of the identified genes varied among different genes. Notably, among all the elements present in the upstream regions of nearly all *CcBAG* genes, light-responsive elements constituted more than half of the identified elements.

### Protein-protein interaction analysis and 3D structure prediction of *CcBAG* proteins

**FIGURE 7:**
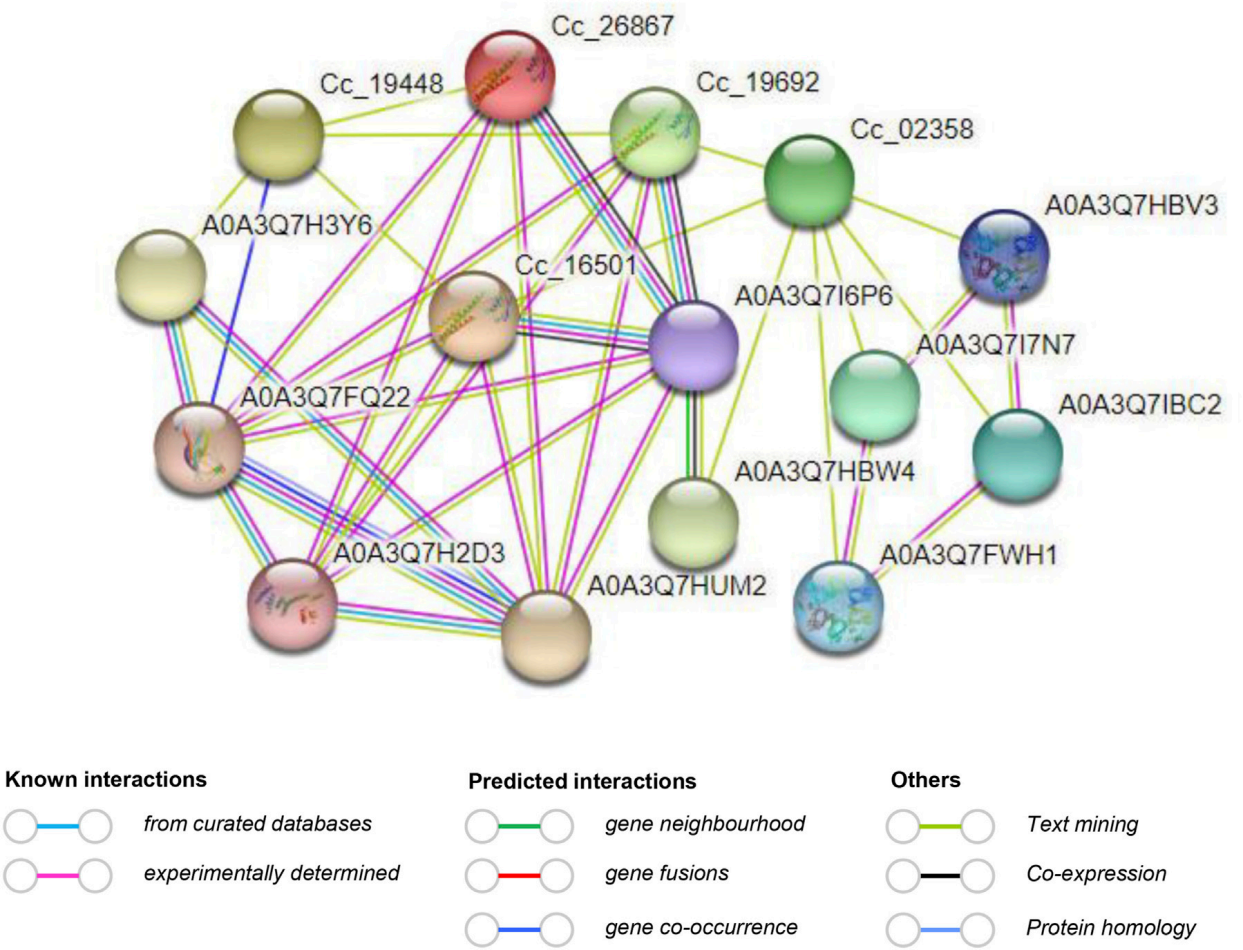
Predicted protein-protein interaction network of *CcBAG* proteins obtained from STRING (https://string-db.org/) database. The red line indicates the presence of fusion evidence; The green line is neighborhood evidence; The blue line is co-occurrence evidence; The purple line is experimental evidence.

The study of protein–protein interactions is crucial as it provides insights into the molecular regulatory network. In this particular investigation, the interaction network of *CcBAG* proteins revealed their potential interactions with multiple proteins. Notably, proteins of *Cc_18023*, *Cc_15012*, *Cc_11905*, and *Cc_01922* did not exhibit interactions with other proteins. On the other hand, proteins of *Cc_19448*, *Cc_26867*, *Cc_19692*, *Cc_02358*, and *Cc_16501* displayed interactions both among themselves and with additional proteins (Figure 7). This interaction network highlighted A0A3Q7I6P6 (HSP70) as the primary functional node with multiple protein interactions. This suggests that the HSP70 protein interacts with the majority of *CcBAG* proteins and is likely activated in response to defense and stress conditions (Supplementary Table S5). The knowledge of protein 3D structure acts as a pre-requisite to understanding the function of a protein, as a small variation in the sequence of protein could lead to greater structural variation than the native protein. The 3D protein structures of the *CcBAG* genes revealed that the *BAG1* (*Cc_18023* and *Cc_26867*), *BAG2* (*Cc_19692*) and *BAG4* (*Cc_15012*, *Cc_11905*, *Cc_01922*, and *Cc_16501*), displayed almost identical structures. The helices, turns/Beta strands and loops were seen very similar in all their structures. The predicted similar structures indicate that these BAG proteins may have potentially similar functions. In contrast, the genes *BAG5* (*Cc_19448*) and *BAG6* (*Cc_02358*) each exhibited significantly different structures with long helices and almost no turns/Beta strands (Supplementary Figure S2).

### GO and miRNA prediction of *CcBAG* genes

The GO enrichment analysis facilitates a better understanding of different functional processes involving *CcBAG* gene family members. It was revealed that all nine *BAG* genes were involved in the cellular anatomical entity (GO: 0005575) of the cellular component category, and binding (GO: 0003674) of the molecular function category (Supplementary Figure S3). We got 7 of the *BAG* gene sequences that are involved in biological regulation (GO:0008150); 3 sequences were identified for cellular process (GO: 0008150) and metabolic process (GO: 0008150); 1 sequence for response to stimulus (GO: 0008150) in biological process category. Similarly, for the molecular function category, 7 of the CcBAG sequences are involved in molecular function regulatory activity (GO: 0003674), and 1 of the sequence for catalytic activity (GO:0003674) were observed. In cellular component category, the intracellular anatomical structure (GO: 0110165) and cytoplasm (GO: 0110165, GO:0005622) were controlled by 7 of the *CcBAG* sequences and 2 of the sequences were shown for the remaining cellular components. The GO enrichment analysis reveals that, out of the 9 CcBAG genes, 7 exhibit similar biological, cellular, and molecular functions, indicating their conserved nature compared to Arabidopsis throughout evolution. In contrast, two BAG genes—likely BAG5 (Cc_19448) and BAG6 (Cc_02358)—show divergence in their biological, cellular, and molecular functions. This divergence is supported by differences in their protein 3-dimensional structures, which may contribute to their altered functions.

The miRNAs control/regulate gene expression at the posttranscriptional level by base pairing with complementary mRNA sequences, leading to gene silencing or degradation of the target mRNA under several stress-related conditions. Hence, the *CcBAG* genes targeted by the miRNAs have been predicted. Out of the nine *BAG* genes, only *Cc_02358* (*BAG6*) gene was found to be targeted by two different miRNA accessions (miRN5120 and miRN5144) with perfect complementarity (Supplementary Figure S4), leading to translational inhibition and silencing of the *BAG* gene irrespective of its higher expression in susceptible genotypes and different protein 3-dimensional structure that facilitates the post translational inhibition.

### Differential expression of *BAG* genes in pigeonpea during heat stress

**FIGURE 8:**
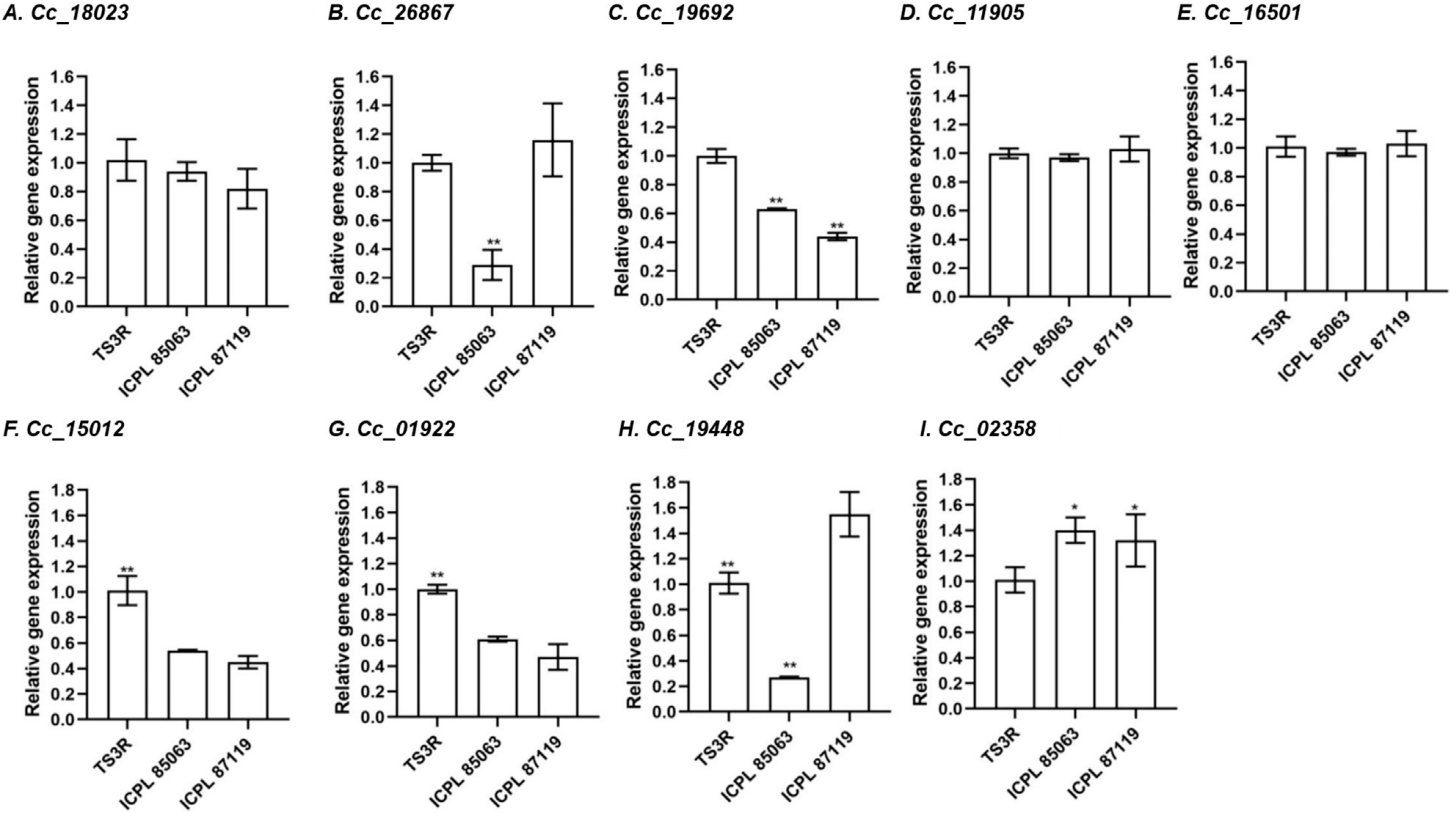
Relative transcript expression in flower tissues of contrasting pigeonpea genotypes during heat stress, based on qRT–PCR in comparison to reference gene *GAPDH*. **(A)**. *Cc_18023* (*BAG1*), **(B)**. *Cc_26867* (*BAG1*), **(C)**. *Cc_19692* (*BAG2*), **(D)**. *Cc_11905* (*BAG4*), **(E)**. *Cc_16501* (*BAG4*), **(F)**. *Cc_15012* (*BAG4*), **(G)**. *Cc_01922* (*BAG4*), **(H)**. *Cc_19448* (*BAG5*), **(I)**. *Cc_02358* (*BAG6*). TS3R is a heat-tolerant genotype; ICPL 85063 and ICPL 87119 are heat-susceptible genotypes. *P< 0.05, **P< 0.01, a significant difference in expression level in tolerant compared to susceptible ones.

To elucidate the expression patterns of the *CcBAG* genes, flower and leaf tissues from diverse pigeonpea genotypes exposed to heat stress were chosen. Following established practices, the *GAPDH* gene, a potentially functional endogenous reference gene commonly utilized for normalization, was selected based on prior studies (Sinha et al., 2015). While all nine *CcBAG* genes were expressed in all the genotypes, it was evident that each *CcBAG* gene exhibited a distinct expression pattern (Figure 8; Supplementary Figure S5).

In flower tissues, the heat-tolerant genotype TS3R exhibited upregulation of four genes, namely *Cc_18023* (*BAG1*), *Cc_19692* (*BAG2*), *Cc_15012*, and *Cc_01922* (*BAG4*) compared to susceptible genotypes. Two genes, *Cc_26867* (*BAG1*) and *Cc_19448* (*BAG5*), were exclusively downregulated in the ICPL 85063 genotype. Conversely, one gene *Cc_02358* (*BAG6*), exhibited upregulation in the susceptible genotypes (ICPL 85063 and ICPL 87119), indicating that this gene, along with *Cc_18023* (*BAG1*), *Cc_19692* (*BAG2*), *Cc_15012* and *Cc_01922* (*BAG4*), might play a crucial role in the regulation of heat stress.

Regarding leaf tissue in flowering plants, the gene *Cc_02358* showed notable upregulation in the heat-tolerant genotype TS3R. Conversely, both *Cc_02358* and *Cc_11905* exhibited significant upregulation in the susceptible genotype ICPL 87119. In contrast, when it comes to leaves of non-flowering plants, all *CcBAG* genes displayed similar expression levels across the three genotypes, regardless of their tolerance or susceptibility to heat stress (Supplementary Figure S5).

## Discussion

The *BAG* gene family represents a set of highly conserved proteins found across all eukaryotes, including plants. These proteins play crucial roles in diverse cellular processes such as stress response, development, and immunity (Huang et al., 2022; Jin et al., 2015; Jiang et al., 2023; Dash and Ghag, 2022). Extensive characterization of the *BAG* gene family has been conducted in model plants like *Arabidopsis* (Doukhanina et al., 2006), rice (Rana et al., 2012), wheat (Ge et al., 2016), and soybean (Wang et al., 2020). However, the *BAG* genes in pigeonpea have not been characterized to date. In this study, we identified the *BAG* genes in pigeonpea, explored their evolutionary relationships, compared their sequence features, and scrutinized their expression patterns in flower and leaf tissues under terminal heat stress.

### Identification, evolution, and characteristics of the *BAG* genes in pigeonpea

Through sequence analysis in the CajanusMine database, we identified nine *BAG* genes in pigeonpea, and these genes are distributed across five chromosomes. Pigeonpea exhibits a smaller number of *BAG* genes compared to other legume plants like soybean (12) and groundnut (10), but a higher count than Medicago (5) and chickpea (6). This observation suggests that in dicots, the BAG protein family may be primarily associated with lineage-specific Whole Genome Duplication (WGD) events (Young et al., 2011; Schmutz et al., 2010). The genes of BAG1, BAG2 and BAG4 featured UBL domain, and BAG6 showed the presence of calmodulin-binding IQ motif that binds to calmodulin (CaM), as reported previously by Doukhanina et al. (2006); Nawkar et al. (2017); Kabbage and Dickman (2008).

A phylogenetic analysis of the BAG protein family, categorizing members into five clades, aligns with similar patterns observed in tomato (Irfan et al., 2021) and soybean (Wang et al., 2020). In contrast, groundnut and *M. truncatula* have reported four clades. These findings suggest that the preservation of specific clades may vary among species due to their distinct evolutionary histories. Moreover, events involving the gain or loss of exons/introns contribute to gene structure divergence and functional differentiation (Xu et al., 2012). Understanding the exon-intron structure is crucial for unraveling the relationships between evolutionary processes and functional divergence (Cao et al., 2016; Moore and Purugganan, 2003; Hurley et al., 2005).

In the pigeonpea *BAG* gene family, the number and length of exons/introns exhibit non-conservation, a trend shared with other species such as *Arabidopsis* (Doukhanina et al., 2006), banana (Dash and Ghag, 2022), rice (Rana et al., 2012), wheat (Ge et al., 2016), and soybean (Wang et al., 2020). These differences in exon/intron characteristics may arise from chromosome rearrangements and fusions, potentially leading to distinct biological functions for these genes.

To systematically explore the overall organization of members and establish connections between proteins, biological functions, and subcellular localization, it is crucial to investigate how proteins are distributed within a cell. Subcellular localization aims to assign proteins, responsible for specific biological functions, to specific cell compartments. For example, all *CcBAG*s predominantly localize in the nucleus, except for *Cc_26867* (*BAG1*) and *Cc_19448* (*BAG5*), which are predicted to be located in the chloroplast. This differs from the reported localization of *Arabidopsis* BAG proteins, where *AtBAG*s 1-3 were found in the cytoplasm and *AtBAG*4 in both the cytoplasm and nucleus (Nawkar et al., 2017). *AtBAG*5 and *AtBAG*6 were reported to localize in the mitochondria and vacuole (Doukhanina et al., 2006). This disparity could be attributed to potential nuclear localization signals that are not deposited in the databases, indicating the existence of such signals in BAG proteins (Irfan et al., 2021).

### Regulation of *BAG* genes in pigeonpea

Promoter regions of the *CcBAG* genes were found to contain putative *cis*-acting elements associated with light, stress, hormones, and development, suggesting potential regulation by various abiotic stress factors and hormones. The identification of conserved stress-responsive *cis*-elements such as Long Terminal Repeats (LTR), MYB binding sequences (MBS), MYB-binding sites, and TC-rich repeats implies that *CcBAG* genes may be subject to stress regulation. This observation aligns with the enrichment of stress-responsive elements like MBS and TC-rich repeats in *Arabidopsis BAG* genes (Doukhanina et al., 2006; Nawkar et al., 2017). The presence of stress-related elements in the upstream regions of *BAG* genes in pigeonpea mirrors the pattern observed in *BAG* genes from other crops like Arabidopsis, rice, soybean, wheat, and tomato. Moreover, successful applications of such information for enhancing stress tolerance in rice and Arabidopsis have been reported (Arif et al., 2021; Ge et al., 2016; Irfan et al., 2021; Wang et al., 2020).

### Regulatory relationship between BAGs and HSPs in pigeonpea

The protein-protein interaction map revealed a network comprising 15 nodes and 37 edges, with an average node degree of 4.93. The genes of *BAG1*, *BAG2,* and *BAG4* interacted with HSP70 protein. A similar result of *A. thaliana AtBAG*4, known to bind with *HSP70*, has been reported to suppress abiotic-induced cell death (Doukhanina et al., 2006). The PPI network further indicated that the BAG proteins which were not directly interacting with *HSP70*, formed complexes with the *CcBAG*s that did interact with *HSP70*. This observation suggests the formation of complexes involving molecular chaperones and signaling molecules. The results imply that *BAG* domains, in regulating stress responses, enable BAG-family proteins to function as adapter molecules by recruiting *HSP70/HSC70* to modulate the activity of target proteins. Similar findings regarding the interaction of BAG proteins with *HSPs* were reported in tomato (Irfan et al., 2021).

### Expression analysis of *BAG* genes in pigeonpea during heat stress

Plants have evolved mechanisms to cope with challenging environmental conditions and withstand various stresses (Song et al., 2019). Previous research has demonstrated the involvement of *BAG* genes in responding to multiple stresses (Jiang et al., 2023). In our investigation of the expression of nine *CcBAG* genes in genotypes with varying responses to heat stress, we utilized qRT-PCR to analyze their expression in flower and leaf tissues. Significant differences were observed in the expression levels of these genes in both flowering and non-flowering leaves. Considering the severe impact of heat stress on the reproductive stage of plants, our focus was primarily on flower tissues. In flower tissues, the heat-tolerant genotype TS3R exhibited upregulation of *BAG4* genes (*Cc_15012* and *Cc_01922*). This finding aligns with a previous study (Doukhanina et al., 2006) where *AtBAG*4 was reported to interact with HSP70 and suppress cell death induced by abiotic stress. A significant upregulation in the expression of *Cc_19448* (*BAG5*) in the susceptible genotype ICPL 87119 was in parallel to an experimental result in tomato where overexpression of *SlBAG9* (a homologous gene of *AtBAG*5) exhibited high sensitivity to heat stress (Ding et al., 2020; Ding et al., 2022). Moreover, *AtBAG*6 is known as a heat-inducible protein (Kang et al., 2006; Li et al., 2017; Fu et al., 2019). Previous research indicated that the *AtBAG*6 gene responds to heat stress, and its mutation can impair plant thermotolerance (Nawkar et al., 2017). In our study, the *CcBAG*6 gene, *Cc_02358*, displayed significant upregulation in the susceptible genotypes. This observation is consistent with a study suggesting that a mutation in the *AtBAG*6 gene enhances basic thermotolerance in plants (Echevarria-Zomeno et al., 2016). However, a single *BAG6* mutation did not confer thermotolerance (Fu et al., 2019). The knockout of *AtBAG*6 significantly improved the heat tolerance of the FES1A mutant (a Nucleotide Exchange Factor that increases molecular chaperone efficiency essential for heat tolerance in Arabidopsis) (Fu et al., 2019). It is also proposed that since *AtBAG*6 has a CaM-binding IQ motif, knocking down this gene may release CaM or CaM-like proteins from the nucleus, altering the dynamics of nuclear calcium signaling to enhance HSP expression in FES1A mutants and improve acquired thermotolerance.

Our miRNA analysis revealed that two of the identified miRNA sequences in pigeonpea perfectly complemented the *CcBAG6* gene (*Cc_02358*) which imply either a decrease or complete loss of the protein encoded by this gene. This could likely account for the significant downregulation of this gene expression as observed in the tolerant genotype TS3R, supported by the expected gene silencing effect via translational inhibition, in miRNA target site prediction analysis.

## Conclusion

In this study, we identified a total of nine pigeonpea *BAG* family genes along with their corresponding proteins. These genes were classified into five clades based on their phylogenetic relationships. The study delved into the gene structure, analysis of conserved domains, and an in-depth exploration of the promoter regions through *in silico* analysis. The findings underscore that these proteins interact with HSP70 proteins and predominantly localize in the nucleus and chloroplast. In these cellular compartments, they have the potential to regulate the activity of target proteins by forming complexes with HSP70 during plant stress responses and development. Moreover, the *CcBAG* genes demonstrated responsiveness to stress, with their expression patterns being modulated under heat stress conditions. The functional characterization of these *CcBAG* genes holds promise for a comprehensive understanding of their interactions with signaling pathways. This knowledge can contribute to precise breeding interventions aimed at developing climate-resilient pigeonpea varieties.

## Data Availability

The original contributions presented in the study are included in the article/Supplementary Material, further inquiries can be directed to the corresponding authors.
