# Supplementary figures and images for: Identification and evaluation of BAG (B-cell lymphoma-2 associated athanogene) family gene expression in pigeonpea (Cajanus cajan) under terminal heat stress
Source: Front Genet. 2024 Nov 14;15:1418380. doi: 10.3389/fgene.2024.1418380 (PMC11602463; doi:10.3389/fgene.2024.1418380)

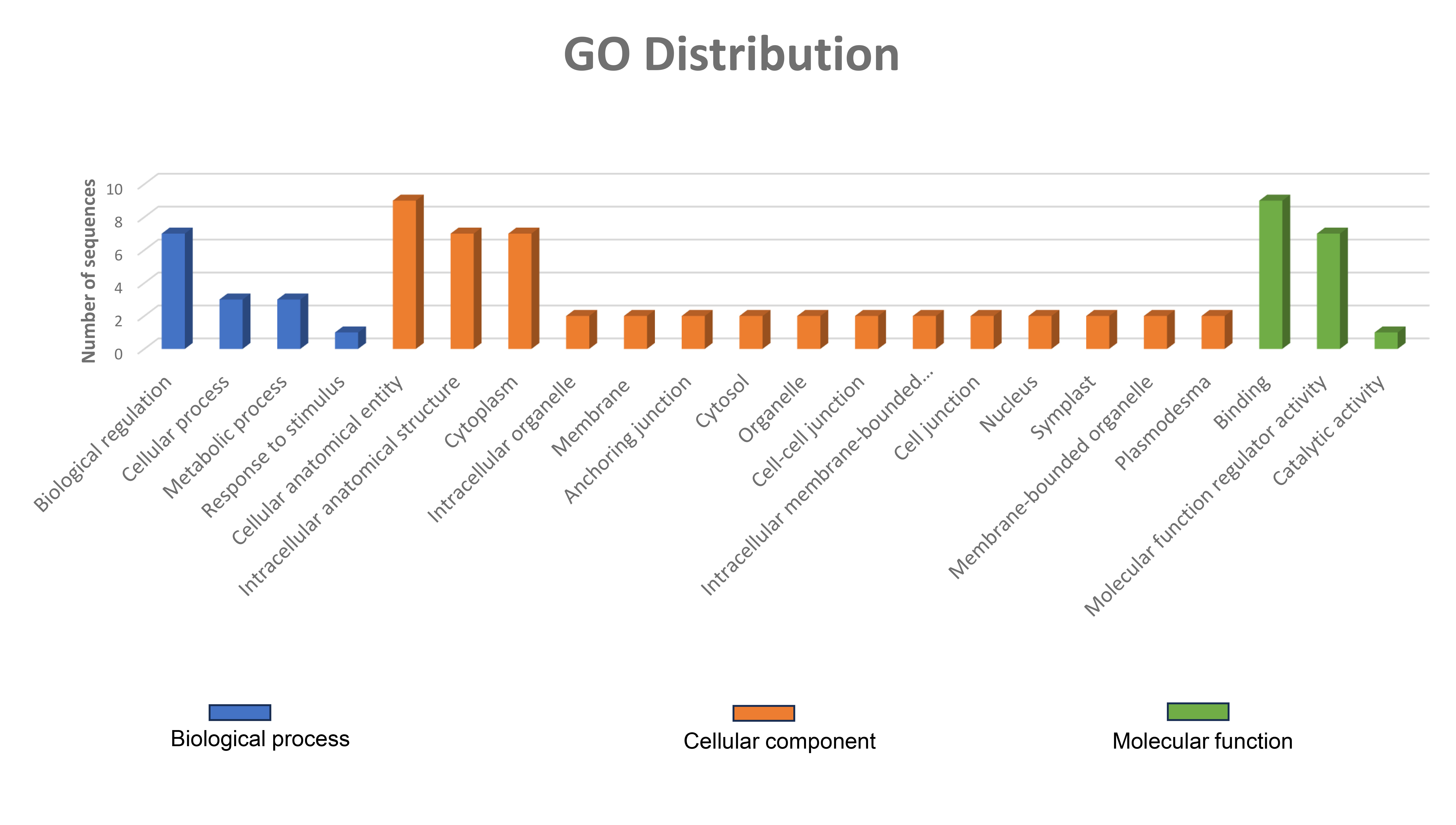

Supplement: Supplementary file 1 [file Image3.jpeg]

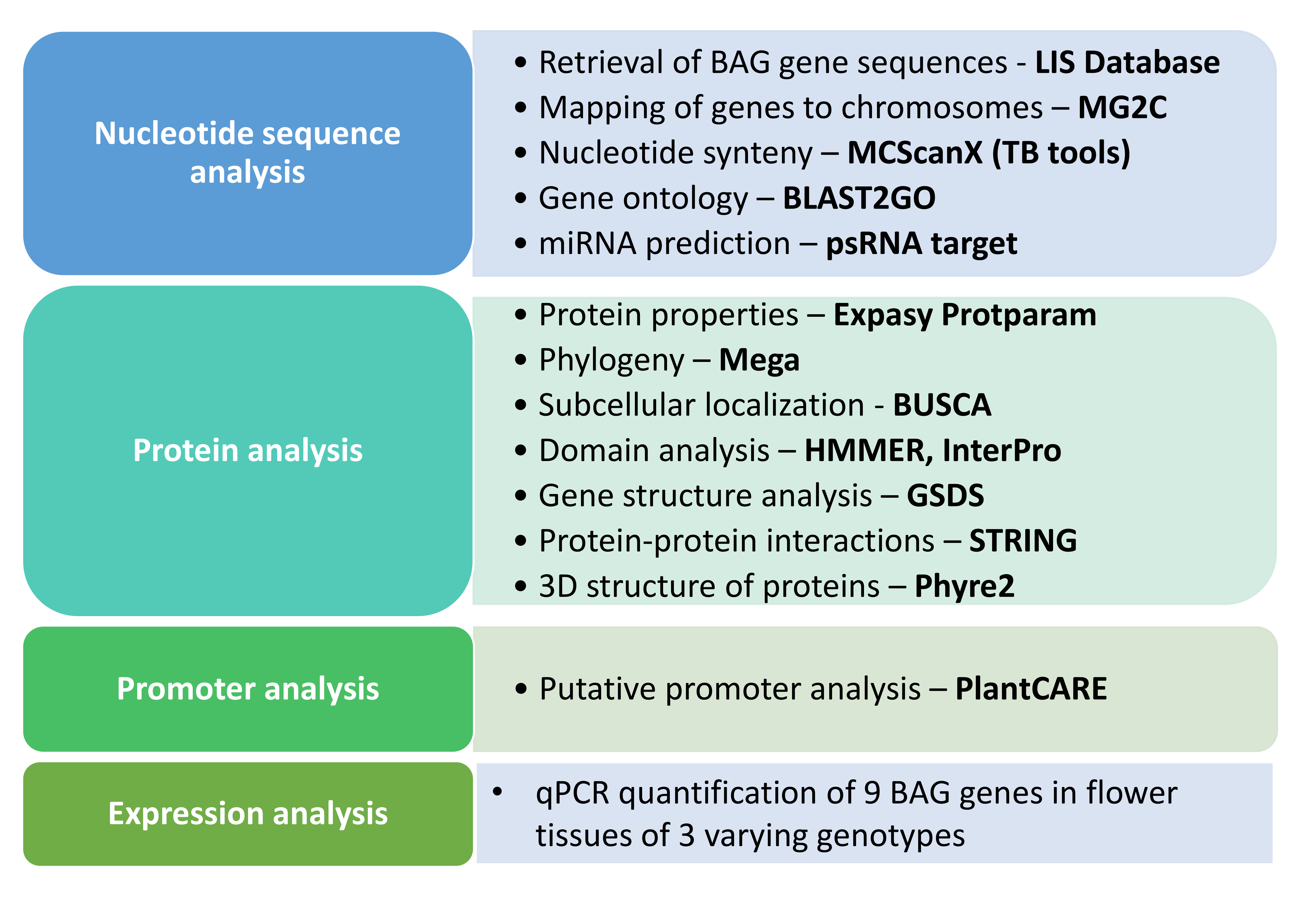

Supplement: Supplementary file 3 [file Image1.jpeg]

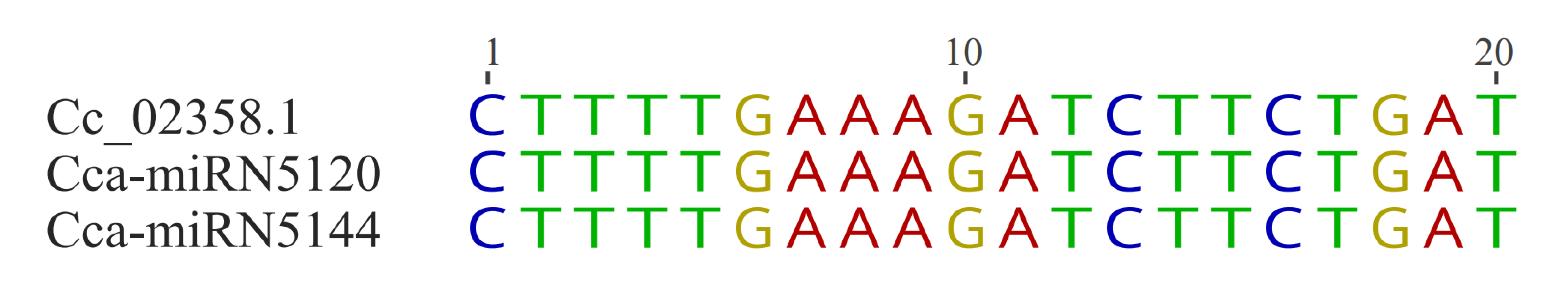

Supplement: Supplementary file 4 [file Image4.jpeg]

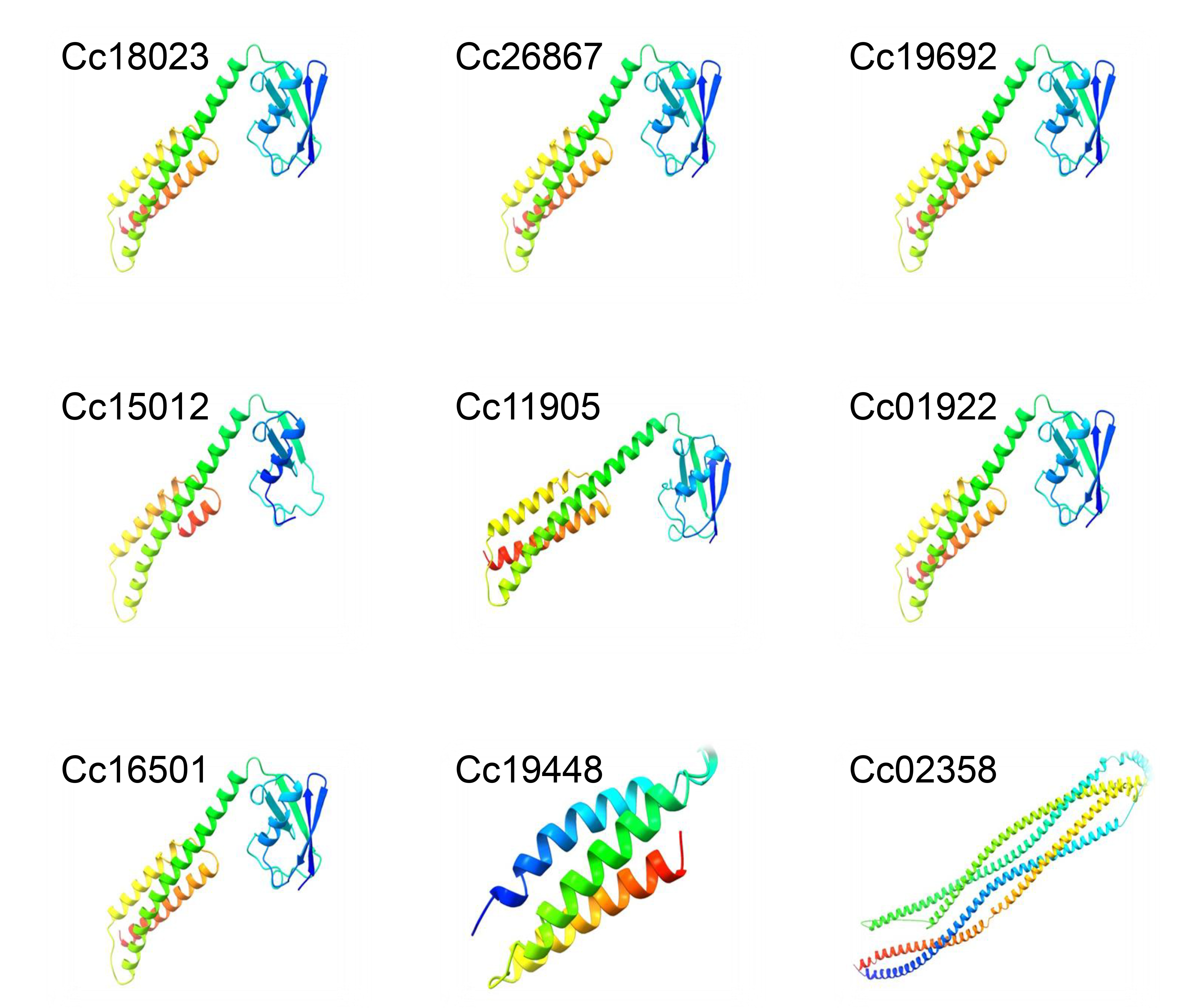

Supplement: Supplementary file 5 [file Image2.jpeg]

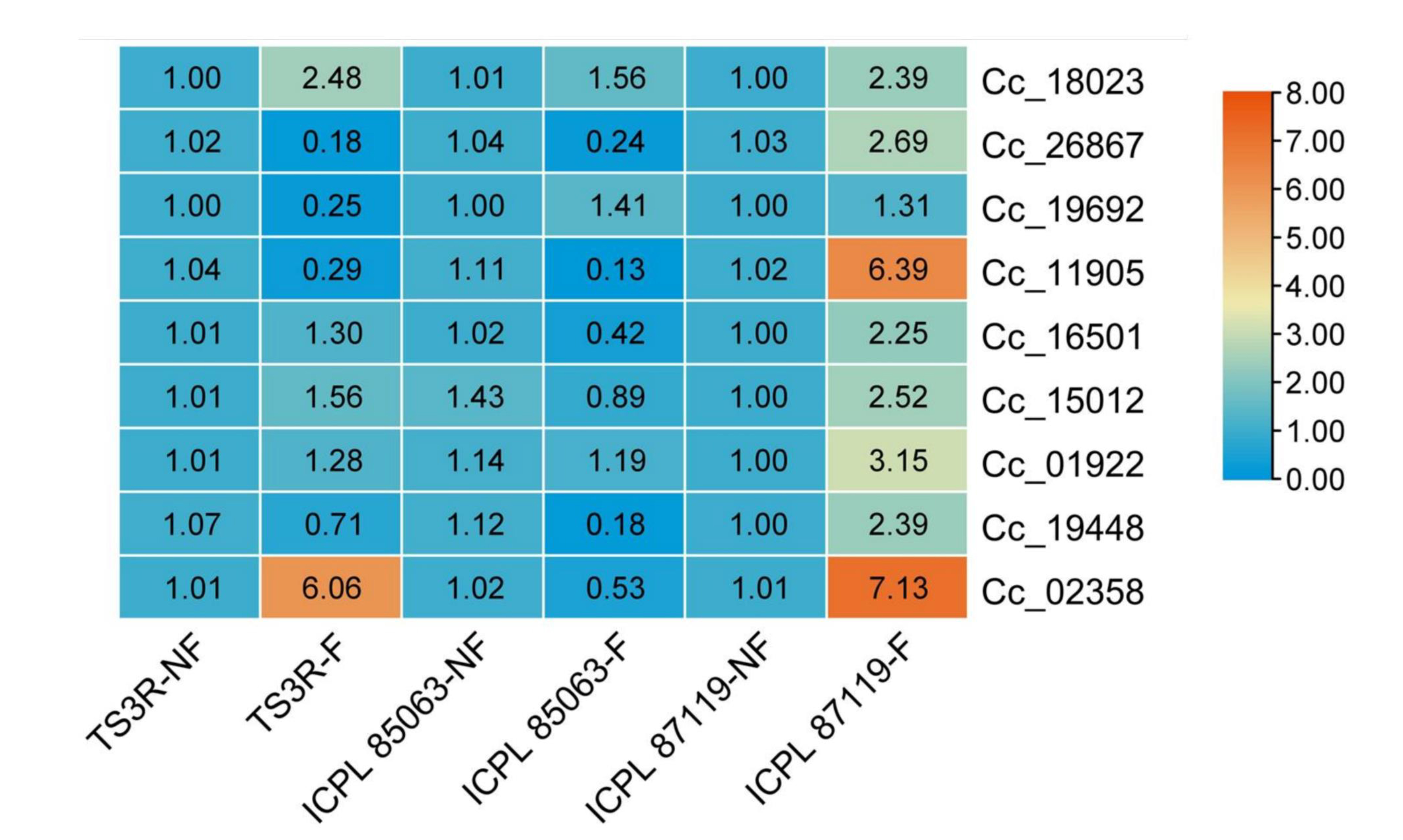

Supplement: Supplementary file 6 [file Image5.jpeg]
