# Supplementary material for: Identification and evaluation of BAG (B-cell lymphoma-2 associated athanogene) family gene expression in pigeonpea (Cajanus cajan) under terminal heat stress
Source: Front Genet. 2024 Nov 14;15:1418380. doi: 10.3389/fgene.2024.1418380 (PMC11602463; doi:10.3389/fgene.2024.1418380)
Supplement: Supplementary file 2 [file Supplementaryfile1.docx]

**Table S1. Daily weather data recorded at ICRISAT, Patancheru (India) during 2023 (Latitude: 17.51°N, Longitude: 78.27°E, Altitude: 545m).**

| **Date** | **Standard week** | **Rain** | **Evaporation** | **Max Temperature** | **Min Temperature** | **Relative Humidity** |
| --- | --- | --- | --- | --- | --- | --- |
| 27-01-2023 | 4 | 0 | 5 | 31.2 | 11.8 | 93 |
| 28-01-2023 | 4 | 0 | 5.2 | 30 | 11.4 | 93 |
| 29-01-2023 | 5 | 0 | 5.2 | 31 | 12.8 | 93 |
| 30-01-2023 | 5 | 0 | 6 | 31 | 19.4 | 80 |
| 31-01-2023 | 5 | 0 | 4.4 | 28.6 | 17.4 | 90 |
| 01-02-2023 | 5 | 0 | 4.2 | 30.2 | 16.2 | 90 |
| 02-02-2023 | 5 | 0 | 5.4 | 30 | 13.6 | 73 |
| 03-02-2023 | 5 | 0 | 5.6 | 30.4 | 10.4 | 86 |
| 04-02-2023 | 5 | 0 | 4.6 | 31 | 11.8 | 84 |
| 05-02-2023 | 6 | 0 | 5.8 | 32.2 | 13.2 | 92 |
| 06-02-2023 | 6 | 0 | 5.8 | 32.2 | 13.2 | 92 |
| 07-02-2023 | 6 | 0 | 5.1 | 32.2 | 16.4 | 84 |
| 08-02-2023 | 6 | 0 | 4.8 | 32.8 | 16.2 | 90 |
| 09-02-2023 | 6 | 0 | 4.4 | 33 | 14.2 | 96 |
| 10-02-2023 | 6 | 0 | 5.4 | 33.2 | 12.4 | 93 |
| 11-02-2023 | 6 | 0 | 5.6 | 33.4 | 11.2 | 86 |
| 12-02-2023 | 7 | 0 | 6 | 34.2 | 12.6 | 91 |
| 13-02-2023 | 7 | 0 | 5.6 | 35 | 12 | 82 |
| 14-02-2023 | 7 | 0 | 5.9 | 33 | 9.6 | 88 |
| 15-02-2023 | 7 | 0 | 6.2 | 33.2 | 9.8 | 88 |
| 16-02-2023 | 7 | 0 | 6.1 | 34.2 | 10.8 | 86 |
| 17-02-2023 | 7 | 0 | 6.3 | 35 | 11.2 | 88 |
| 18-02-2023 | 7 | 0 | 6 | 34.2 | 11.6 | 82 |
| 19-02-2023 | 8 | 0 | 6.4 | 33.2 | 15.4 | 92 |
| 20-02-2023 | 8 | 0 | 5.8 | 33.8 | 15 | 90 |
| 21-02-2023 | 8 | 0 | 6.2 | 33.2 | 14.6 | 94 |
| 22-02-2023 | 8 | 0 | 6.1 | 35.4 | 13.4 | 89 |
| 23-02-2023 | 8 | 0 | 6.2 | 35.8 | 14 | 85 |
| 24-02-2023 | 8 | 0 | 6.6 | 35.2 | 14.2 | 89 |
| 25-02-2023 | 8 | 0 | 6.8 | 35.4 | 13.6 | 87 |
| 26-02-2023 | 9 | 0 | 7.3 | 35.4 | 13.6 | 79 |
| 27-02-2023 | 9 | 0 | 7.5 | 35.2 | 14.8 | 77 |
| 28-02-2023 | 9 | 0 | 7.6 | 34.2 | 16.8 | 82 |
| 01-03-2023 | 9 | 0 | 7.8 | 33.2 | 16.4 | 75 |
| 02-03-2023 | 9 | 0 | 8 | 33.4 | 15.6 | 84 |
| 03-03-2023 | 9 | 0 | 7.2 | 34 | 16.4 | 57 |
| 04-03-2023 | 9 | 0 | 7.2 | 34 | 16.4 | 57 |
| 05-03-2023 | 10 | 0 | 7.4 | 34.8 | 15.6 | 80 |
| 06-03-2023 | 10 | 0 | 7 | 35 | 16 | 78 |
| 07-03-2023 | 10 | 0 | 7.6 | 34.6 | 18.8 | 66 |
| 08-03-2023 | 10 | 0 | 8 | 35.4 | 14.2 | 83 |
| 09-03-2023 | 10 | 0 | 7.2 | 33.8 | 15.2 | 80 |
| 10-03-2023 | 10 | 0 | 7.6 | 33.8 | 15.4 | 86 |
| 11-03-2023 | 10 | 0 | 6 | 34.4 | 16 | 74 |
| 12-03-2023 | 11 | 0 | 6.6 | 33.4 | 18.4 | 85 |
| 13-03-2023 | 11 | 0 | 6.9 | 34 | 15.4 | 88 |
| 14-03-2023 | 11 | 0 | 7.1 | 34.8 | 16.6 | 73 |
| 15-03-2023 | 11 | 0 | 7.7 | 35.2 | 15.4 | 76 |
| 16-03-2023 | 11 | 0 | 7 | 35.2 | 19 | 84 |
| 17-03-2023 | 11 | 15.4 | 4 | 30.2 | 19.2 | 93 |
| 18-03-2023 | 11 | 9.4 | 6 | 27.6 | 18.6 | 96 |
| 19-03-2023 | 12 | 52.4 | 5.2 | 30.4 | 16.4 | 96 |
| 20-03-2023 | 12 | 0 | 5 | 30.2 | 18.6 | 91 |
| 21-03-2023 | 12 | 0 | 5.2 | 33 | 16 | 78 |
| 22-03-2023 | 12 | 0 | 5.6 | 31.6 | 17 | 79 |
| 23-03-2023 | 12 | 0 | 5.8 | 33.4 | 17.2 | 89 |
| 24-03-2023 | 12 | 0 | 5.6 | 34.6 | 18.6 | 93 |
| 25-03-2023 | 12 | 0 | 5.8 | 35.2 | 20.8 | 83 |
| 26-03-2023 | 13 | 0 | 5.6 | 35 | 19.8 | 86 |
| 27-03-2023 | 13 | 0 | 6 | 36.6 | 20.2 | 84 |
| 28-03-2023 | 13 | 0 | 6.2 | 36.6 | 19.2 | 81 |
| 29-03-2023 | 13 | 0 | 6.9 | 37.4 | 21.4 | 85 |
| 30-03-2023 | 13 | 0 | 7 | 37.4 | 20.6 | 75 |
| 31-03-2023 | 13 | 0 | 7.2 | 37.8 | 21.2 | 70 |
| 01-04-2023 | 13 | 0 | 6 | 34.6 | 18.6 | 74 |
| 02-04-2023 | 14 | 0 | 6.2 | 34.6 | 18.6 | 82 |
| 03-04-2023 | 14 | 0 | 5.8 | 35.2 | 22.4 | 80 |
| 04-04-2023 | 14 | 0 | 7.2 | 37.2 | 21.4 | 73 |
| 05-04-2023 | 14 | 0 | 7.5 | 37.4 | 20.6 | 84 |
| 06-04-2023 | 14 | 0 | 8.3 | 38.6 | 23.4 | 85 |
| 07-04-2023 | 14 | 0 | 7 | 35.4 | 21.8 | 71 |
| 08-04-2023 | 14 | 0 | 7.2 | 35.2 | 22.6 | 67 |
| 09-04-2023 | 15 | 0 | 7.6 | 35.2 | 18.6 | 76 |
| 10-04-2023 | 15 | 0 | 7.6 | 36.4 | 18.6 | 75 |
| 11-04-2023 | 15 | 0 | 7.8 | 37 | 19 | 80 |
| 12-04-2023 | 15 | 0 | 7.9 | 38 | 20.4 | 65 |
| 13-04-2023 | 15 | 0 | 8.2 | 38.4 | 22.6 | 62 |
| 14-04-2023 | 15 | 6 | 7.6 | 39 | 21.4 | 83 |
| 15-04-2023 | 15 | 0 | 6 | 37.8 | 21.8 | 69 |
| 16-04-2023 | 16 | 0 | 6.1 | 36.2 | 21.2 | 70 |
| 17-04-2023 | 16 | 0 | 7.6 | 38 | 21.4 | 72 |
| 18-04-2023 | 16 | 0 | 7.8 | 39 | 19.6 | 82 |
| 19-04-2023 | 16 | 0 | 9 | 40.2 | 22 | 57 |
| 20-04-2023 | 16 | 0 | 9.4 | 40.2 | 24.6 | 40 |
| 21-04-2023 | 16 | 0 | 8.4 | 39.4 | 25 | 59 |
| 22-04-2023 | 16 | 4.2 | 7.9 | 36.2 | 21.6 | 78 |
| 23-04-2023 | 17 | 0 | 7.8 | 37.6 | 19.6 | 85 |
| 24-04-2023 | 17 | 0 | 7.2 | 37.4 | 23 | 78 |
| 25-04-2023 | 17 | 0 | 7.5 | 37.4 | 23.6 | 81 |
| 26-04-2023 | 17 | 109.2 | 7.6 | 36.2 | 18.4 | 96 |
| 27-04-2023 | 17 | 0 | 4 | 34.2 | 22.4 | 86 |
| 28-04-2023 | 17 | 0 | 5.3 | 33.4 | 23 | 90 |
| 29-04-2023 | 17 | 32.4 | 6 | 34.8 | 19.2 | 96 |
| 30-04-2023 | 18 | 3.2 | 5.4 | 33.2 | 23.2 | 79 |
| 01-05-2023 | 18 | 8.2 | 4.3 | 32.8 | 20.6 | 91 |
| 02-05-2023 | 18 | 0 | 5 | 31 | 22.6 | 82 |
| 03-05-2023 | 18 | 0 | 5.2 | 31.4 | 22 | 88 |
| 04-05-2023 | 18 | 0 | 4.4 | 32 | 22.4 | 88 |
| 05-05-2023 | 18 | 3.4 | 4.6 | 33.2 | 21.8 | 84 |
| 06-05-2023 | 18 | 0 | 5.4 | 33.2 | 23.2 | 87 |
| 07-05-2023 | 19 | 0 | 5.2 | 34 | 22.6 | 88 |
| 08-05-2023 | 19 | 0 | 6 | 34.8 | 24.2 | 83 |
| 09-05-2023 | 19 | 0 | 6.6 | 36.4 | 24.6 | 86 |
| 10-05-2023 | 19 | 14.2 | 5.8 | 35.6 | 20.4 | 92 |
| 11-05-2023 | 19 | 0 | 6.2 | 36.2 | 25.2 | 83 |
| 12-05-2023 | 19 | 0 | 5.9 | 36.4 | 22.6 | 77 |
| 13-05-2023 | 19 | 0 | 6 | 37.4 | 21.4 | 77 |
| 14-05-2023 | 20 | 0 | 6.4 | 38.8 | 22.8 | 69 |
| 15-05-2023 | 20 | 0 | 6.9 | 39 | 25.2 | 63 |
| 16-05-2023 | 20 | 0 | 7.6 | 38.4 | 21 | 74 |
| 17-05-2023 | 20 | 0 | 7.4 | 38 | 20.2 | 77 |
| 18-05-2023 | 20 | 0 | 7 | 38.4 | 22.4 | 56 |
| 19-05-2023 | 20 | 0 | 7.2 | 39.2 | 22.6 | 75 |
| 20-05-2023 | 20 | 0 | 7.4 | 39.4 | 24.4 | 67 |
| 21-05-2023 | 21 | 0 | 7.6 | 40 | 22.4 | 74 |
| 22-05-2023 | 21 | 30.2 | 8.4 | 40.2 | 20.2 | 98 |
| 23-05-2023 | 21 | 0 | 4 | 37.4 | 23.6 | 84 |
| 24-05-2023 | 21 | 0 | 5.2 | 37.2 | 23 | 85 |
| 25-05-2023 | 21 | 0 | 6.2 | 38.2 | 24.8 | 69 |
| 26-05-2023 | 21 | 0 | 6 | 35.4 | 25.2 | 64 |
| 27-05-2023 | 21 | 0 | 5.8 | 37 | 24.4 | 68 |
| 28-05-2023 | 22 | 0 | 6 | 38.4 | 21.8 | 67 |
| 29-05-2023 | 22 | 0 | 6.6 | 38 | 24.2 | 66 |
| 30-05-2023 | 22 | 0 | 6.8 | 38.2 | 25.2 | 64 |
| 31-05-2023 | 22 | 0 | 7 | 35 | 24.4 | 77 |
| 01-06-2023 | 22 | 0 | 7 | 39 | 22.6 | 78 |
| 02-06-2023 | 22 | 0 | 7.6 | 40 | 25.4 | 64 |
| 03-06-2023 | 22 | 0 | 7.9 | 40 | 26.2 | 53 |
| 04-06-2023 | 23 | 0 | 7.4 | 40.2 | 27.2 | 57 |
| 05-06-2023 | 23 | 22.8 | 6.4 | 37.8 | 20.6 | 91 |
| 06-06-2023 | 23 | 0 | 7 | 36 | 23.4 | 71 |
| 07-06-2023 | 23 | 0 | 6.8 | 38.4 | 25.2 | 61 |
| 08-06-2023 | 23 | 0 | 7.2 | 38.4 | 25.2 | 68 |
| 09-06-2023 | 23 | 0 | 6.4 | 38 | 24.2 | 70 |
| 10-06-2023 | 23 | 0 | 7.2 | 38.6 | 25.4 | 70 |
| 11-06-2023 | 24 | 0 | 6.6 | 37.6 | 24.4 | 70 |
| 12-06-2023 | 24 | 0 | 6.8 | 36.6 | 25.4 | 69 |
| 13-06-2023 | 24 | 0 | 7.2 | 38.2 | 24.8 | 66 |
| 14-06-2023 | 24 | 0 | 7.6 | 38.2 | 25.4 | 66 |
| 15-06-2023 | 24 | 0 | 7.9 | 38.2 | 25.2 | 65 |
| 16-06-2023 | 24 | 0 | 8.2 | 38.4 | 25.6 | 61 |
| 17-06-2023 | 24 | 0 | 8.6 | 38.2 | 25.6 | 66 |
| 18-06-2023 | 25 | 0 | 8.6 | 38.2 | 25.6 | 66 |
| 19-06-2023 | 25 | 0 | 8.2 | 38.2 | 27.2 | 59 |
| 20-06-2023 | 25 | 0 | 8.4 | 37.4 | 24.8 | 60 |
| 21-06-2023 | 25 | 0 | 7.8 | 39.4 | 25.2 | 62 |
| 22-06-2023 | 25 | 34.8 | 9.4 | 38.4 | 23.2 | 73 |
| 23-06-2023 | 25 | 0 | 5 | 35.2 | 24.2 | 70 |
| 24-06-2023 | 25 | 52.6 | 8.4 | 34.8 | 21.6 | 88 |
| 25-06-2023 | 26 | 24.8 | 5.6 | 31.2 | 22.4 | 88 |
| 26-06-2023 | 26 | 0 | 4 | 31.6 | 23.6 | 85 |
| 27-06-2023 | 26 | 0 | 3.6 | 31.2 | 22.8 | 81 |
| 28-06-2023 | 26 | 5.4 | 3.9 | 30.4 | 23.2 | 82 |
| 29-06-2023 | 26 | 0 | 3.7 | 32.2 | 22.8 | 81 |
| 30-06-2023 | 26 | 0 | 3.8 | 32.4 | 22.6 | 81 |

**Table S2: List of primers used for the expression analysis of BAG genes in pigeonpea**

| **Gene ID** | **annotation** | **qPCR primers** | **sequences** | **Product size** |
| --- | --- | --- | --- | --- |
| Cc_18023 | BAG1 | Cc_18023_qPCR-FP | CTCAGTGCTTGTTCCCACTAT | 172 bp |
|  |  | Cc_18023_qPCR-RP | TGAATCCCTCTCCTTGTCTTTG |  |
| Cc_26867 | BAG1 | Cc_26867_qPCR-FP | CACAAACGTGGCTGAATTGG | 159 bp |
|  |  | Cc_26867_qPCR-RP | AAACTTGCATGAGAGCTGATTTG |  |
| Cc_19692 | BAG2 | Cc_19692_qPCR_FP | GTTTACAGGTGGATGAGCAGAG | 178 bp |
|  |  | Cc_19692_qPCR_RP | CGATGTGCGGTTTGGATCTTA |  |
| Cc_15012 | BAG4 | Cc_15012_qPCR_FP | TGGACAAGCTCGCTGAAA | 145 bp |
|  |  | Cc_15012_qPCR_RP | GCTTCACCTTCAGCTTCAATAC |  |
| Cc_11905 | BAG4 | Cc_11905_qPCR_FP | CCAGTGTCAGAACAGAAGTAGAC | 163bp |
|  |  | Cc_11905_qPCR_RP | GCTTCTCCCTCAGCAGAAAT |  |
| Cc_01922 | BAG4 | Cc_01922_qPCR_FP | CAAATAGGGTGACTGCCATAGA | 123 bp |
|  |  | Cc_01922_qPCR_RP | CAGCCTCAATACCATCCAGTT |  |
| Cc_16501 | BAG4 | Cc_16501_qPCR_FP | GATGTCCACGGAGTTGCTTAT | 160bp |
|  |  | Cc_16501_qPCR_RP | GCTGGTATAAGGGTTGGAGTTT |  |
| Cc_19448 | BAG5 | Cc_19448_qPCR_FP | CCGACATGGAGGAGACTATTTG | 181bp |
|  |  | Cc_19448_qPCR_RP | GCTACCCTACACTCTCTGACT |  |
| Cc_02358 | BAG6 | Cc_02358_qPCR_FP | TGTTCCAGTGAAGTCTCATGTT | 175bp |
|  |  | Cc_02358_qPCR_RP | GTGATCTCCCTCCGATTCATTT |  |
| CcGAPDH |  | Forward primer | GGTGGTGCAAAGAAGGTTATCA | 183bp |
|  |  | Reverse primer | CAAACCCTCAACAATGCCAAAC |  |

**Table S3: Detailed information BAG genes of Pigeonpea corresponding gene ID, coding sequences, chromosome location, number of exons, subcellular localization, and predicted physiochemical properties.**

| **SI. No.** | **BAG domain** | **Gene ID** | **CDS length** | **Amino acid length** | **Location** | **No. of exons** | **Localization** | **Mol. Weight (in kDa)** | **Theoretical pI** | **Aliphatic index** |
| --- | --- | --- | --- | --- | --- | --- | --- | --- | --- | --- |
| 1 | BAG1 | Cc_18023 | 1089 | 362 | 8 | 4 | Nucleus | 40.21 | 9.5 | 74.25 |
| 2 |  | Cc_26867 | 853 | 279 | 11 | 4 | Chloroplast | 31.60 | 9.63 | 84.16 |
| 3 | BAG2 | Cc_19692 | 768 | 251 | 8 | 4 | Nucleus | 28.18 | 9.11 | 85.42 |
| 4 | BAG4 | Cc_15012 | 527 | 172 | 7 | 4 | Nucleus | 19.41 | 4.95 | 100.93 |
| 5 |  | Cc_11905 | 805 | 263 | 5 | 4 | Nucleus | 29.57 | 7.02 | 86.27 |
| 6 |  | Cc_01922 | 777 | 254 | 1 | 4 | Nucleus | 28.05 | 5.82 | 74.96 |
| 7 |  | Cc_16501 | 762 | 249 | 7 | 4 | Nucleus | 28.21 | 5.69 | 79.08 |
| 8 | BAG5 | Cc_19448 | 597 | 195 | 8 | 2 | Chloroplast | 21.75 | 5.31 | 101.59 |
| 9 | BAG6 | Cc_02358 | 3528 | 1156 | 1 | 2 | Nucleus | 129.75 | 5.00 | 55.47 |

**Table S4: Ka/Ks value representing average Ka/Ks ratios of collinear gene pairs of BAG genes from *Cajanus cajan* and *Medicago truncatula*.**

| **Seq_1** | **Seq_2** | **Ka** | **Ks** | **Ka/Ks** |
| --- | --- | --- | --- | --- |
| Cc_18023.1 | MtrunA17Chr6g0473621.1 | 0.107349331 | 0.690113265 | 0.155553206 |
| Cc_01922.1 | MtrunA17Chr7g0243051.1 | 0.221995647 | 0.681102961 | 0.325935518 |
| Cc_15012.1 | MtrunA17Chr7g0243051.1 | 0.22743227 | 0.502730766 | 0.452393777 |
| Cc_16501.1 | MtrunA17Chr7g0243051.1 | 0.185189596 | 0.57746965 | 0.320691478 |
| Cc_19448.1 | MtrunA17Chr2g0289801.1 | 0.159219525 | 2.0590783 | 0.077325629 |
| Cc_02358.1 | MtrunA17Chr4g0018451.1 | 0.845285422 | 3.676125892 | 0.229939193 |

**Table S5: Annotations of Proteins used for protein-protein interactions using string software.**

| **Node** | **Domain summary** | **Annotation** |
| --- | --- | --- |
| A0A3Q7FQ22 | Uncharacterized protein | Next to the BRCA1 gene one protein |
| A0A3Q7FWH1 | Germin-like protein | Auxin-binding protein ABP19a |
| A0A3Q7H2D3 | Uncharacterized protein | A0A3Q7H2D3_SOLLC |
| A0A3Q7H3Y6 | Uncharacterized protein | Uncharacterized protein |
| A0A3Q7HBV3 | Germin-like protein | Auxin-binding protein ABP19a |
| A0A3Q7HBW4 | Uncharacterized protein | A0A3Q7HBW4 |
| A0A3Q7HUM2 | ZZ-type domain-containing protein | Next to the BRCA1 gene one protein |
| A0A3Q7I6P6 | Uncharacterized protein | Heat shock 70 kDa protein 17 |
| A0A3Q7I7N7 | AP2/ERF domain-containing protein | AP2/ERF domain-containing protein |
| A0A3Q7IBC2 | AP2/ERF domain-containing protein | AP2/ERF domain-containing protein |
| Cc_02358 | BAG domain-containing protein | BAG domain-containing protein |
| Cc_16501 | Uncharacterized protein | BAG family molecular chaperone regulator 4 |
| Cc_19448 | BAG domain-containing protein | BAG domain-containing protein |
| Cc_19692 | Uncharacterized protein | BAG family molecular chaperone regulator 3 |
| Cc_26867 | Uncharacterized protein | BAG family molecular chaperone regulator 3-like |
